# Factors associated with adherence to provider referrals for lung cancer screening with low dose computed tomography before and during COVID-19 pandemic

**DOI:** 10.1186/s12885-023-11256-9

**Published:** 2023-08-29

**Authors:** Jiang Li, Cheryl D. Stults, Su-Ying Liang, Meghan Martinez

**Affiliations:** Palo Alto Medical Foundation Research Institute, Center for Health Systems Research, Sutter Health, 795 El Camino Real, 94301 Palo Alto, CA USA

**Keywords:** Tobacco-related disease, Longitudinal research, Early detection, Completion rate, Multilevel analysis

## Abstract

**Background:**

Lung cancer has been the leading cause of American deaths from cancer. Although Medicare started covering lung cancer screening (LCS) with low-dose computed tomography (LDCT) in 2015, the uptake of LDCT-LCS remains low. This study examines the changes in adherence to provider referrals for LDCT-LCS and the factors at patient, provider, and health system levels that influence the completion rate of LDCT-LCS orders before and during the COVID-19 pandemic.

**Methods:**

Our study examined electronic health record data (December 2013 - December 2020) from a large, community-based clinical healthcare delivery system in California. We plotted monthly trends in the frequency of LDCT-LCS orders and completion rate and compared the annual LDCT-LCS completion rate between LCS-eligible, LCS-ineligible, and unknown eligibility groups. We then explored multilevel factors associated with the completion of LDCT-LCS orders using hierarchical generalized linear models.

**Results:**

There was an increase in LDCT-LCS orders (N = 12,469) from 2013 to 2019, followed by a sharp decline in March 2020 due to the onset of the COVID-19 pandemic. Thereafter, LDCT-LCS orders slowly increased again in June 2020. The completion rate of LDCT-LCS increased from 0% in December 2013 to approximately 70% in 2018–2019 but declined to 50–60% in 2020 during the pandemic. Ineligible patients had lower completion rates of LDCT-LCS. Patients who were new to the healthcare system, Black, received the LDCT-LCS order in the first few years after Medicare coverage (2016 or 2017), during the pandemic, had major comorbidities, and smoked less than 30 pack-years were less likely to complete an order. Patients were more likely to complete LDCT-LCS orders if they were younger, received the LDCT-LCS order from a physician (vs. nonphysician provider), from family medicine or other specialties (vs. internal medicine), or saw a provider with more experience in LDCT-LCS.

**Conclusions:**

The beginning of the COVID-19 pandemic largely decreased the volume of LDCT-LCS orders, but rates have since been slowing recovering. Future interventions to improve lung cancer screening should consider doing more targeted outreach to new patients and Black patients as well as providing additional education to nonphysician practitioners and those providers with lower rates of LDCT-LCS referral orders.

## Background

Lung cancer is the leading cause of death from cancer in the U.S [1]. Most lung cancer is caught late in the disease process with 5-year survival rates as low as 6%, but can be improved to over 60% if detected in early stages [2]. Lung cancer screening (LCS) with low dose computed tomography (LDCT) has shown a 20% relative reduction in lung cancer mortality compared to chest X-rays in individuals with high lung cancer risk [3]. Given this, the U.S. Preventive Services Task Force (USPSTF) and other organizations have recommended LDCT-LCS for people who heavily smoke since December 2013 [4–10] and it has been covered by Medicare since February 2015 [11]. With reductions in smoking and improvements in early detection and treatment [2], the annual decline in lung cancer mortality has doubled from 2.4% before LDCT-LCS guideline changes (2009–2013) to 5% after guideline changes (2014–2018).

Despite coverage, LDCT-LCS referrals among eligible patients remains low, with estimates ranging from 3.9 to 7.3%, and ineligible individuals continue to be referred for LDCT-LCS[12]. Many barriers exist for both patients and providers, such as time constraints, cost, lack of awareness, and lack of trust in healthcare systems, and these can impede utilization of LDCT-LCS in clinical settings [13–15]. Moreover, LCS involves multiple components, many of which are unique among preventive cancer screenings: determining eligibility, shared decision making (SDM), placing the referral, smoking cessation counseling, screening, interpreting results, and managing follow-ups according to multidisciplinary protocols [16–20]. In March 2021, the USPSTF updated guidance for LDCT-LCS to include populations at lower absolute risk for lung cancer (adults aged 50 to 80 years who have a 20 pack-year smoking history and currently smoke or have quit within the past 15 years), which makes millions more people who smoke eligible for discussions about screening and smoking cessation [21].

Coronavirus disease 2019 (COVID-19) has further exacerbated the already low rates of LDCT-LCS by changing the risk/ benefit of cancer screening with the risk of COVID-19 infection [22]. From April 2020 to August 2020, the American Cancer Society (ACS) issued recommendations that no one should go to a healthcare facility for routine (nondiagnostic) cancer screening due to the added risks from potential exposure to COVID-19 and the need for resource reallocation [23]. Some research has attempted to examine the effects of the COVID-19 pandemic on LCS. While 19 states experienced significant improvements in LCS rates among eligible adults despite the COVID-19 pandemic, California had the lowest rates in the country (1.4% in 2019 and 1.1% in 2020) [24]. Our study aims to further contribute to the literature to assess trends in adherence to referrals for LDCT-LCS among LCS-eligible, LCS-ineligible, and unknown eligibility patients before and during the COVID-19 pandemic in a large ambulatory care organization in central and northern California and to examine the multilevel factors influencing completion rates of LDCT-LCS orders which could inform ongoing efforts to detect lung cancers early.

## Methods

### Study sample and data sources

Sutter Health is a large community healthcare setting in central and northern California that serves a diverse patient population (covering urban and rural) with varying insurance types. We used electronic health records (EHRs) between December 2013 and December 2020 to identify all patients who smoke aged 55–80 who had at least one office visit during a given year and were referred for LDCT-LCS. Adult primary care at Sutter Health is delivered by providers who specialize in internal medicine or family medicine, collectively referred to as primary care providers (PCPs). The EHR data were linked longitudinally at the patient encounter level, and include billing, diagnosis, procedures, clinical encounter records, and provider notes (text fields).

Using the linked data, we looked back in time before LDCT-LCS to identify details of care, including previous screening patterns, as well as activities during that period that may be relevant to understanding the circumstances surrounding the screening decisions.

We also included additional provider and clinic characteristics such as sex, profession (physician or nonphysician practitioner), and prior experience with LDCT-LCS defined as the number of patients with LDCT-LCS order above or below mean.

LCS-eligible patients were defined as adults ages 55 to 80 with at least a 30 pack-year smoking history and who currently smoke or have quit within the past 15 years. A pack year is the number of packs smoked per day multiplied by the number of years (e.g., 1 pack/day for 30 years equals 30 pack years). Patients with unknown pack years or quit years were classified as an unknown eligibility group.

### Measures

A LDCT-LCS order completed within 12 months of the order date and before the next available LDCT-LCS order is defined as a completed LDCT-LCS order. In the first part of the study, a patient-month was the unit of analysis. We examined each month separately, so a patient could appear in the denominator (and numerator) in multiple months. In the overall study sample (12,469 patient years over 2013–2020) of patients who received one or more order(s) of LCS with LDCT, we calculated the frequency of LDCT-LCS orders and percentage of completed LDCT-LCS orders each month to describe the adherence to provider referrals for LDCT-LCS among LCS-eligible and LCS-ineligible patients as well as patients with unknown eligibility. The number of LDCT-LCS orders and proportion of completed orders (completion rate) during 2013–2020 was the dependent variable. The key independent variable (time) is an indicator variable for calendar years and months. The frequency and completion rate of LDCT orders was compared before (i.e., 2013–2019) and during (i.e., 2020) the COVID-19 pandemic.

For multilevel analysis, we focused on orders between 2016 and 2020 because Medicare coverage was released on October 15th, 2015 for claims with dates of service retroactive to February 5th, 2015. The unit of analysis is the patient. Dependent variable (defined at patient level) is the completion of first LDCT-LCS order (Yes/No) among those who received one or more order(s) of LCS with LDCT during 2016–2020.

Individual-level independent variables include time, age (55–64, 65–77, 78–80), sex, race/ethnicity (White, Black, Hispanic, Asian, other), smoking status (currently, formerly, never), smoking history (pack-years), severity of major comorbidities, whether visiting own PCP, types of health insurance (private or self-pay, public), department (family medicine, internal medicine, pulmonary, other), and type of visit when the LDCT-LCS order was placed (Medicare wellness, health maintenance exam, new patient appointment, established patient appointment, other). For the severity of major comorbidities, Charlson Comorbidity Index (CCI) [25] was used and patients were divided into four groups: no major comorbidity, with CCI scores of 0; mild, with CCI scores of 1–2; moderate, with CCI scores of 3–4; and severe, with CCI scores ≥ 5. A patient’s own PCP refers to the PCP whom the patient actively chose when first visiting a PCP and usually goes to when he or she needs preventive services. Provider-level independent variables include provider gender, profession (physician vs. other clinician), and prior experience of practices derived from the EHR (e.g., number of patients with a LDCT-LCS referral in the panel when the LDCT-LCS order was placed).

### Statistical analysis

First, we plotted monthly trends between 2013 and 2020 in the frequency of LDCT-LCS orders and completion rate. We compared the annual LDCT-LCS completion rate between LCS-eligible, LCS-ineligible, and unknown eligibility groups by calendar year using Chi-square tests. Among those who had their first LDCT order in 2016–2020 and one or more office visits within one year after the LDCT order, we conducted bivariate analysis and unconditional tests of association between patient characteristics and completion of LDCT-LCS order using Chi-square tests. We then used hierarchical generalized linear models (HGLM) with a logit link function for binary outcome, with covariates including time as the key predictor and sociodemographic variables such as age, sex, race/ethnicity, and smoking status as the potential confounders. Data were analyzed using a multilevel structure with patients (level 1) nested within providers (level 2). In order to avoid within-patient clustering, we only included a patient’s first LDCT-LCS order. The relative importance in the variation in each level was determined by intraclass correlation (ICC) apportioning the variance in the outcome across patient and provider levels (i.e., correlation between the patients within the same provider). ICC in the case of logistic regression was computed using the linear threshold model method, or latent variable method supported by Snijders and Bosker [26].

Following a model-building strategy as discussed by Raudenbush and Bryk [27], we ran a series of HGLM to obtain the point estimates for the parameters of interest including the odd ratios for the predictors and the random effect variance. We fitted multilevel random intercepts models in a stepwise fashion: Model 1 (unconditional model) is a 2-level null model including no fixed effects; Model 2 (patient model) includes only patient-level variables; and Model 3 (final model) includes both patient-level and provider-level variables. Coefficients from Models 2 and 3 were compared to test whether variance in outcome can be explained by patient and provider factors. Tests of hypotheses for the fixed effects are based on Wald-type tests and the estimated variance-covariance matrix. The nested models were compared using a Likelihood Ratio Test. Statistical analyses were performed using SAS Version 9.4. This study was reviewed and a Waiver of Informed Consent was granted by the Sutter Health Institutional Review Board (SHIRB # 2018.023EXP), and all methods were conducted in accordance with the Declaration of Helsinki. This study was approved by the Sutter Health Institutional Review Board.

## Results

**Fig. 1:**
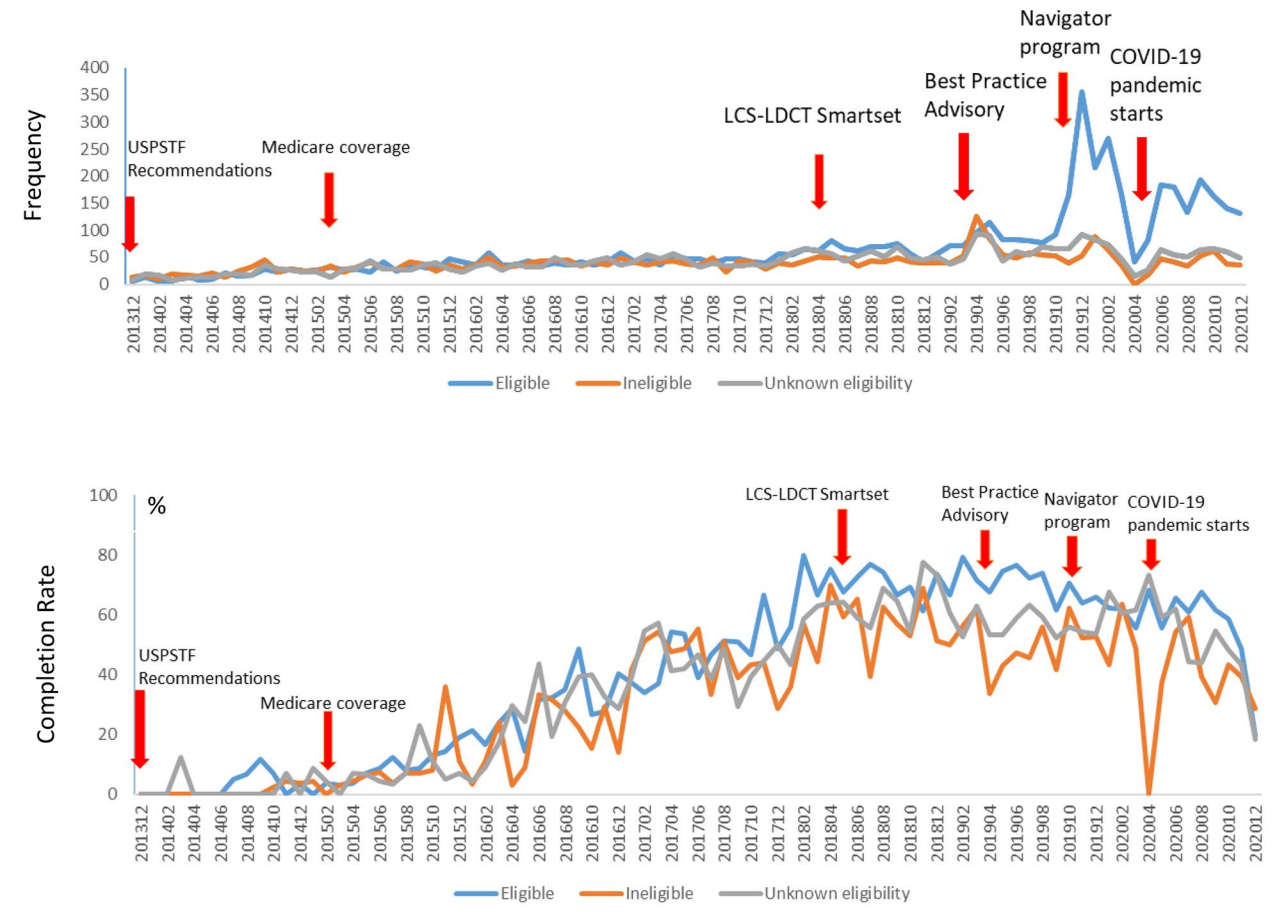
Trends in frequency and completion rate of LDCT orders (Dec.2013 - Dec. 2020)

Among patients aged 55 and 80 years who had at least one office visit during 2013 to 2020 at a Sutter Health facility, monthly total number of LDCT orders increased from 26 in December 2013 to 499 in December 2019. LDCT orders dropped dramatically at the start of COVID-19 pandemic to 56 in April 2020, then slowly went up to 200–300 every month (Fig. 1). Among patients aged 55 to 80 years who received LDCT orders, the proportion of patients who were eligible and completed LDCT-LCS increased from 0% in December 2013 to around 70% in 2018–2019 and then declined to 50–60% in 2020 during COVID-19 pandemic. Of the ineligible patients and patients with unknown eligibility, the completion rates of LDCT-LCS were lower than that of the eligible patients, with the difference being even more prominent after 2018 (unknown eligibility vs. ineligible vs. eligible in 2018: 62.2% vs. 56.1% vs. 70.0%, p < 0.0001; 2019: 56.2% vs. 48.2% vs. 69.3%, p < 0.0001; 2020: 52.8% vs., 45.5% vs. 57.7%, p < 0.0001).

Of the 4,939 patients who had their first LDCT order in 2016–2020 and one or more office visits within the 12 months after the LDCT order, a total of 2,860 (57.9%) had completed LDCT orders within 12 months (Table 1). Bivariate results show that the percent completing LDCT-LCS orders varied significantly between Asian (53.4%), Hispanic (55.9%), Non-Hispanic White (59.1%), Black (43.3%), and other racial/ethnic groups (60.6%) (p < 0.0001). Patients who were younger (i.e., 55–64 years old and 65–77 years old, compared to 78–80 years old) (p < 0.0001), had public insurance (Medicare or Medicaid) (p < 0.05), or had smoked 30 or more pack-years (p < 0.0001) were significantly more likely to complete LDCT-LCS. Completion rates also vary across type of visit, department, and year (all p < 0.0001) (Table 1).

**Table 1:** Characteristics of patients with LDCT-LCS orders between 2016 and 2020

	Completion of LDCT-LCS Order						P value
	No (N = 2079)		Yes (N = 2860)		All(N = 4939)		
	N	%	N	%	N	%	
**Age Group**							< 0.0001
55–64	881	46	1034	54	1915	38.8	
65–77	1121	38.8	1770	61.2	2891	58.5	
78–80	77	57.9	56	42.1	133	2.7	
**Sex**							0.6119
Female	961	42.3	1312	57.7	2273	46.0	
Male	1118	41.9	1548	58.1	2666	54.0	
**Race/Ethnicity**							< 0.0001
Hispanic	124	44.1	157	55.9	281	5.7	
Black	122	56.7	93	43.3	215	4.4	
Asian	138	46.6	158	53.4	296	6.0	
Other	63	39.4	97	60.6	160	3.2	
White	1632	40.9	2355	59.1	3987	80.7	
**Severity of major comorbidities**							0.06909
Severe: CCI > = 5	173	45.8	205	54.2	378	7.7	
Moderate: CCI = 3–4	262	40.2	389	59.8	651	13.2	
Mild: CCI = 1–2	865	40.6	1263	59.4	2128	43.1	
No comorbidity: CCI = 0	779	43.7	1003	56.3	1782	36.1	
**Smoking status**							0.60559
Formerly smoked	1044	41.6	1468	58.4	2512	50.9	
Currently smokes	1035	42.6	1392	57.4	2427	49.1	
**Insurance**							0.01025
Private or self	799	44.8	986	55.2	1785	36.1	
Public	1280	40.6	1874	59.4	3154	63.9	
**Pack-year (packs per day × years of smoking)**							< 0.0001
Less than 30 pack-year	739	49.8	746	50.2	1485	30.1	
30 + pack-year	1340	38.8	2114	61.2	3454	69.9	
**Visit to own primary care provider**							0.25496
Yes	1620	42.5	2193	57.5	3813	77.2	
No	459	40.8	667	59.2	1126	22.8	
**Type of visit when the LDCT-LCS order was placed**							< 0.0001
Medicare Wellness	129	33.1	261	66.9	390	7.9	
Health Maintenance Exam	402	41.3	571	58.7	973	19.7	
New Patient	334	48.8	351	51.2	685	13.9	
Established Patient	1152	41.7	1612	58.3	2764	56.0	
Other	62	48.8	65	51.2	127	2.6	
**Department where the LDCT-LCS order was placed**							< 0.0001
Family Medicine	1237	41.7	1732	58.3	2969	60.1	
Internal Medicine	660	46.5	759	53.5	1419	28.7	
Pulmonary	127	33.2	256	66.8	383	7.8	
Other	55	32.7	113	67.3	168	3.4	
**Year**							< 0.0001
2020	618	42.7	828	57.3	1446	29.3	
2019	494	36.5	861	63.5	1355	27.4	
2018	285	32.7	586	67.3	871	17.6	
2017	304	44.6	378	55.4	682	13.8	
2016	378	64.6	207	35.4	585	11.8	

**Table 2:** Patient- and Provider-level factors affecting LDCT-LCS completion, 2016–2020

Fixed Effects	Model 1	Model 2	Model 3^a^
	Estimate (SE)	Estimate (SE)	Estimate (SE)
Intercept	0.25(0.06)	-0.12(0.29)	-0.8(0.34)
**Patient Level**		**OR (95% CI)**	**OR (95% CI)**
**Age**			
55–64		1.76(1.12–2.77)*	1.81(1.15–2.85)**
65–77		2.48(1.61–3.8)***	2.52(1.64–3.86)***
78–80		1	1
**Sex**			
Female		0.98(0.84–1.13)	0.98(0.85–1.14)
Male		1	1
**Race/Ethnicity**			
Hispanic		0.88(0.65–1.2)	0.87(0.64–1.18)
Black		0.63(0.44–0.9)*	0.64(0.45–0.91)*
Asian		0.95(0.69–1.31)	0.97(0.7–1.33)
Other		1.22(0.82–1.81)	1.21(0.82–1.79)
White		1	1
**Smoking status**			
Formerly Smoked		0.87(0.75–1.01)	0.87(0.75-1)
Currently Smokes		1	1
**Pack-year (packs per day × years of smoking)**			
< 30		0.57(0.49–0.68)***	0.57(0.48–0.67)***
30+		1	1
**Severity of major comorbidities**			
Severe (CCI ≥ 5)		0.73(0.55–0.98)*	0.73(0.55–0.98)*
Moderate (CCI = 3–4)		0.97(0.77–1.23)	0.97(0.77–1.22)
Mild (CCI = 1–2)		1.08(0.92–1.27)	1.08(0.92–1.28)
No major comorbidity (CCI = 0)		1	1
**Health Insurance**			
Private or self		0.91(0.76–1.1)	0.91(0.75–1.09)
Public		1	1
**Type of visit when the LCS-LDCT order was placed**			
Medicare Wellness		1.32(0.99–1.76)	1.31(0.99–1.75)
Health Maintenance Exam		1.12(0.91–1.36)	1.12(0.91–1.37)
New Patient		0.79(0.63–0.99)*	0.78(0.62–0.98)*
Other		0.81(0.51–1.28)	0.81(0.51–1.28)
Established Patient		1	1
**Visit to own primary care provider**			
Yes		1.03(0.8–1.33)	0.85(0.64–1.11)
No		1	1
**Year**			
2020		0.66(0.54–0.8)***	0.67(0.55–0.81)***
2018		1.2(0.95–1.51)	1.18(0.94–1.49)
2017		0.52(0.41–0.67)***	0.52(0.4–0.66)***
2016		0.18(0.14–0.23)***	0.17(0.13–0.22)***
2019		1	1
**Department where the LCS-LDCT order was placed**			
Family Medicine		1.45(1.12–1.87)**	1.46(1.13–1.88)**
Pulmonary		2.3(1.21–4.37)*	1.77(0.93–3.37)
Other		2.23(1.25–3.98)**	2.71(1.48–4.95)**
Internal Medicine		1	1
**Provider Level**			
**Sex**			
Female			0.92(0.73–1.18)
Male			1
**Professional**			
Physician			1.52(1.05–2.21)*
Other			1
**Prior experience**			
Number of patients with LCS-LDCT order above mean			1.87(1.36–2.57)**
Number of patients with LCS-LDCT order below mean			
**Error Variance**	**Estimate (SE)**	**Estimate (SE)**	**Estimate (SE)**
Level-2 Intercept	1.33 (0.15)***	1.46 (0.17)***	1.39 (0.16)***
**Model Fit**			
-2 Log Likelihood	6249.48	5893.82***	5869.69***

Note: ICC = 0.29;

OR Odds Ratio, SE Standard Error, 95% CI 95% Confidence interval

*p < 0.05; ** p < 0.01; ***p < 0.0001

a: Best fitting model

**Fig. 2:**
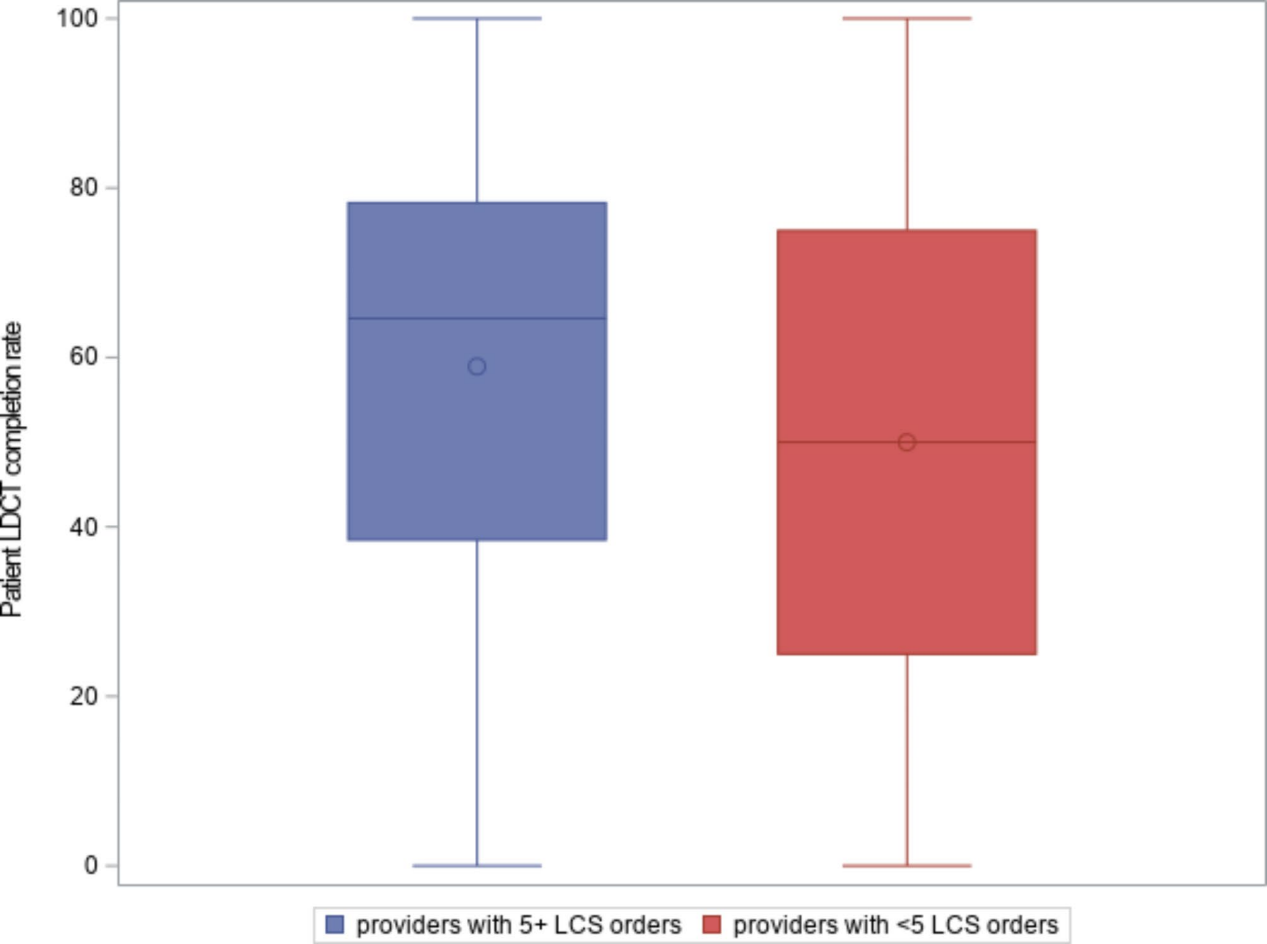
Distribution of patient completion rates of lung cancer screening among 946 providers, 2016–2020

Of the 946 PCPs, 60.3% were female, 82.2% were physicians, and 65% had greater prior experience (i.e., number of patients with LDCT-LCS order above mean). There was substantial variation in completion rates across providers in this healthcare system (Fig. 2). A majority of providers had a completion rate of 50% or more during the five years after Medicare coverage was implemented. While the providers who referred 5 or more patients between 2016 and 2020 had an average completion rate of 59%, the providers who referred fewer than 5 patients had an average completion rate of 50%.

Results from the HGLM (Table 2) also demonstrate the probability of completing LDCT varies considerably across providers, as indicated by statistically significant Level-2 intercept (variance of provider-level trajectory intercepts = 1.33, SE = 0.15, z(945) = 8.58, p < 0.0001) of the unconditional, null model (Model 1). Using data from 4,939 unique patients nested within 946 providers, the probability of LDCT completion by a typical health care provider is 0.563. We computed the intraclass correlation coefficient (ICC = 0.29) that indicates approximately 29% of the variability in the LDCT completion rate is accounted for by the providers in our study, leaving 71% of the variability to be accounted for by the patients or other unknown factors.

Based on the likelihood ratio test, Model 2 with inclusion of the patient-level independent variables was a better fitting model than the unconditional, null model (Model 1). The addition of specific provider-level variables (Model 3) further improved model fit. In this final model, patients aged 55–64 years old (OR = 1.81; 95% CI:1.15–2.85) or 65–77 years old (OR = 2.52; 95% CI:1.64–3.86) were significantly more likely to complete lung cancer screening orders compared to those aged 78–80 years old. Black patients (OR = 0.64; 95% CI:0.45–0.91), patients with severe major comorbidities (OR = 0.73; 95% CI:0.55–0.98), or those who smoked less than 30 pack-years (OR = 0.57; 95% CI:0.48–0.67) were significantly less likely to complete an order than Non-Hispanic Whites, those without any major comorbidity, or those who smoked 30 pack-years or more. New patients who received an LDCT-LCS order were less likely to complete LDCT-LCS orders than the established patients (OR = 0.78; 95% CI:0.62–0.98). Compared to 2019, patients receiving their first LDCT-LCS order in 2016 (OR = 0.17; 95% CI:0.13–0.22), 2017 (OR = 0.52; 95% CI:0.40–0.66), or 2020 (OR = 0.67; 95% CI: 0.55–0.81) were less like to complete the LDCT-LCS order. Patients who received the LDCT-LCS order at the Family Medicine department (OR = 1.46; 95% CI:1.13–1.88) or other specialities (OR = 2.71; 95% CI:1.48–4.95), or from physicians (OR = 1.52; 95% CI: 1.05–2.21) were more likely to complete the order than those visited Internal Medicine or received an order from a nonphysician practitioner. Patients seeing a provider who ordered more than the average number of LDCT were also significantly more likely to complete the order (OR = 1.87; 95% CI:1.36–2.57).

## Discussion

To the best of our knowledge, this is the first study to explore the comprehensive picture of challenges and supports surrounding LDCT-LCS from a multilevel, multifactor perspective in a large community healthcare system in the context of the COVID-19 pandemic. We found that those who received orders in Family Medicine or other specialties, had no major comorbidities, smoked at least 30 pack-years, and saw providers having more experience in LDCT-LCS ordering were more likely to complete an order, highlighting opportunities to improve LDCT-LCS processes and influence clinical practice at patient, provider, and systems levels.

In our data, orders for LDCT-LCS increased since the USPSTF recommendations, decreased substantially at the start of COVID-19 pandemic, then slowly went up, which is in correspondence with the ACS recommendations in April 2020 to postpone cancer screenings in order to prioritize urgent medical needs and reduce the risk of the spread of COVID-19 in health care settings [28]. However, while we found that the absolute number of LDCT-LCS orders declined dramatically at the beginning of the COVID-19 pandemic in 2020, the completion rate only slightly declined and remained at 50–60% throughout 2020. The patients receiving LDCT-LCS orders in 2019 and early 2020 might have postponed the screening test due to the COVID-19 pandemic, but a majority still completed LDCT-LCS within a year. Lang et al. found a similar trend in which institutional LDCT-LCS volume based on lung imaging data significantly decreased during the COVID-19 pandemic, followed by complete recovery of follow-up LDCT-LCS volume and more gradual recovery of annual and baseline LCS LDCT volume [29]. While many patients who did receive LDCT-LCS screening did complete it, there are still a large number who postponed their screenings. Healthcare systems should similarly examine surveillance data for cancer screening orders and completion rates to identify areas to enhance public communications, inform policy changes, and guide interventions as the cancer screenings that were missed or delayed because of pandemic could potentially lead to more patients with more advanced stages at diagnosis, poorer survival, and greater disease-related and treatment-related morbidity.

It is worth noting that before 2019, LCS-ineligible patients and patients with unknown eligibility received referrals for LDCT-LCS no less frequently than LCS-eligible patients, which may be the result of poor documentation of smoking history or that many clinicians and patients might not have been fully aware of the eligibility criteria for LDCT-LCS [30–32]. Unlike other types of cancer screening that recommend universal screening in healthy populations of a certain age, LDCT-LCS targets older people who have smoked heavily long-term, determined using age, pack-years, and current smoking status. Likely exacerbating this at the provider level, identification of these LCS-eligible patients is hindered by the lack of complete and accurate information about smoking history at the point of care [33]. In our data, those with unknown pack years or quit years were separated as the unknown eligibility group, which enables us to reveal the issue of poor documentation of the smoking history among patients with LDCT-LCS orders. Recently in March 2021 USPSTF expanded screening eligibility for individuals at lower absolute risk for lung cancer by lowering screening start age to 50 years and smoking history to 20 pack-years. This change has increased the relative percentage of individuals eligible for screening compared with the 2013 criteria [34], and now includes more racial ethnic minorities and women [35]. With this expansion, millions more people who smoke will become eligible for the LDCT-LCS and the orders for LDCT-LCS among ineligible patients may likely further decline. If LCS-ineligible patients and patients with unknown eligibility continue to be referred for screening under the expanded guidelines, it may warrant further examination to understand whether this is driven by patients or providers and the reasons behind these referrals.

Among patients aged 55 to 80 years who received LDCT orders, the completion rates of eligible patients increased from 0% to 2013 to the highest monthly rate of 70% in 2019, indicating increased lung cancer screening awareness and acceptance in the 5 years since the USPSTF recommendations. Furthermore, LCS-eligible patients had a higher completion rate than the LCS-ineligible patients. Changes in organization-level workflows that may have played an important role in this include a Sutter-wide EPIC system change (“Smartset”) in April 2018, the Best Practice Advisory (BPA) for LDCT-LCS in place in April 2019, and implementation of a Navigator Program for LDCT-LCS in October 2019. The EHR-based LDCT-LCS multidisciplinary management system (“Smartset”) includes “structured tobacco history” allowing for identification of eligible patients, “order workflow” facilitating LCS orders for eligible patients during office visits, and “tracking workflow” enabling Lung Cancer Care Coordinators/Navigators to track qualifying patients through reporting worklists. These system-level changes may provide more coordinated, integrated care that leads to informed decision-making and improved patient adherence.

While we found that our completion rate among patients who had their first LDCT order in 2016–2020 was consistent with the adherence rates from previous studies [36, 37], we also found substantial variation across providers in completion of LDCT-LCS orders. When it comes to completion of LDCT-LCS orders, each clinic has their own processes, systems, and workflows, and there is no unified way to schedule, track, or ensure patients complete screenings or follow-ups. In our study, higher completion rates were found for more experienced providers who ordered more than the average number of LDCT-LCS; however, even after taking into account provider sex, profession, and prior experience, we observed large, unexplained variation across providers, suggesting the need for system-wide efforts to standardize LDCT-LCS practice [38].

With under a third of the variability in the LDCT completion rate accounted for by providers, this leaves a majority of the variability coming from patient factors or other unknown factors. At the patient level, we found that older age (78–80 years old), self-identifying as Black, having severe major comorbidities, having smoked fewer than 30 pack-years, being a new patient, and receiving LDCT-LCS orders in earlier years (i.e., 2016, 2017) or during the COVID-19 pandemic (i.e.,2020) were negative predictors of completing LDCT-LCS orders. The maximum age limit of 77 set by CMS for reimbursement of annual LDCT for lung cancer screening [39] may in part explain the lower completion rates among the oldest age group (78–80 years old), as well as the decreasing relative benefit of screening and early detection with increasing age and severity of comorbidities. Similarly, the previous eligibility criteria of at least 30 pack-years [39, 40] may explain the lower completion rates among those who smoked fewer than 30 pack-years and thus were considered ineligible for LDCT-LCS at the time of this study. Recently expanded USPSTF screening criteria are expected to improve completion rates among those who smoked between 20 and 29 pack-years and enable earlier detection of lung cancer and improved survival outcomes [41]. Consistent with a previous study examining the racial difference in lung cancer screening outcomes [36], we also found that Black patients had significantly lower odds of receiving LDCT compared to White patients even after controlling for other covariates. The changes in 2021 screening guidelines have been associated with a very small difference in lung cancer screening eligibility between Black and White individuals of − 12.7 percentage points in 2013 and − 12.2 percentage points in 2021 [42]. This finding along with the findings of our study suggest that accounting for factors beyond lowering the recommended ages and pack-years for LDCT-LCS is likely needed to significantly decrease disparities in access to lung cancer screening and treatment among racial and ethnic minority groups.

Additionally, we found that receiving the LDCT-LCS order in Family Medicine or other specialties (as opposed to the internal medicine department) and from physicians (as opposed to other providers) were positive predictors of completing LDCT-LCS orders. This is consistent with the findings of a recent study on a Safety-Net Medical System which also found that family practice providers ordered more LDCT-LCS than did other clinicians [38]. Further research is warranted to investigate the high completion rates for LDCT-LCS orders received in Family Medicine and other specialties. While internists typically diagnose and treat medical problems of greater complexity than family practitioners in both the office and hospital settings, family practitioners typically provide preventive medicine and more “well-patient” services in the office setting [43]. This likely results in a difference in provider’s capacity to provide support services following a LDCT-LCS order and continuity in the sense of overall responsibility to ensure patient’s completion of the order.

Our patient-level findings suggest that certain groups of patients – such as those who are older, racial ethnic minority, new to the system, have severe major comorbidities and receive LDCT-LCS order during COVID-19 pandemic may face additional barriers found in other studies like inconvenience, perceived smoking-related stigma, and distrust of the healthcare system which may lead to decreased LCS participation [44]. Health systems should consider focusing on improving these areas as they may potentially increase the likelihood that LDCT-LCS counseling and follow up will take place.

Several limitations to this study need to be acknowledged. We relied on structured data from billing, procedure, ordering, and administrative records, but many important constructs remain unexamined. For example, we lack information on specific factors influencing patient and provider decision-making including SDM weighing pros and cons and patients’ preferences, which may be in part available in unstructured, free-text EHR notes. Similarly, since the organization is not a health maintenance organization (HMO), it is difficult to know whether a patient completed the LDCT-LCS order outside the system. To address this limitation, we selected patients who had one or more office visits within one year after the LDCT order. If they had later office visits at Sutter Health, the care they received elsewhere prior to the visits will be available in our EHR system by the Care Everywhere function. Furthermore, using data from a single healthcare organization with a highly-insured patient population may limit the generalizability of our findings. However, Sutter’s population is about 10% of the state of California [45]. It reflects the diversity that makes up the state and includes low-income, rural, non-English speaking, and minority patients. One benefit of studying a single system is that its shared “infrastructure” allows us to control for access barriers and instead focus on variation across patients and providers in completion of LDCT-LCS orders within the healthcare system.

## Conclusions

Seven years after the USPSTF recommendations and nearly six years after Medicare coverage, the number of orders for LDCT-LCS has increased tremendously. The beginning of the COVID-19 pandemic largely decreased the volume of LDCT-LCS orders, but it has been slowing recovering. Additionally, changes in policies and procedures of implementing LDCT-LCS program by both Medicare and Sutter Health appear to play an important role in LDCT-LCS orders and completion rates. There is wide variation across providers in our healthcare organization in referrals for LDCT-LCS, suggesting the need for system-wide efforts to facilitate appropriate adherence to LDCT-LCS. Future interventions to improve lung cancer screening should consider doing more targeted outreach to new patients and Black patients as well as providing additional education to nonphysician practitioners and those providers with lower rates of LDCT-LCS referral orders.

## Data Availability

The datasets generated and/or analyzed during the current study are not publicly available due to compliance with Health Insurance Portability and Accountability Act of 1996 (HIPAA). Please contact Dr. Li (lij14@sutterhealth.org) if someone wants to request the data from this study.

## Abbreviations

LDCT-LCS: Low-Dose Computed Tomography for Lung Cancer Screening
USPSTF: US Preventive Services Task Force
SDM: Shared Decision Making
COVID-19: Coronavirus Disease 2019
ACS: American Cancer Society
EHR: Electronic Health Records
PCP: Primary Care Provider
CCI: Charlson Comorbidity Index
HGLM: Hierarchical Generalized Linear Models
ICC: Intra-class Correlation Coefficient
OR: Odds Ratio
SE: Standard Error
95% CI: 95% Confidence Interval
HMO: Health Maintenance Organization
BPA: Best Practice Advisory
